# Contemporary Approach to Acute Pancreatitis in Emergency Medicine

**DOI:** 10.1016/j.acepjo.2025.100063

**Published:** 2025-02-18

**Authors:** Kegham Hawatian, Munir Sidani, Thomas Hagerman, Shaun Condon, Christine Chien, Joseph Miller

**Affiliations:** 1 Department of Emergency Medicine, Henry Ford Hospital, Henry Ford Health and Michigan State University Health Sciences, Detroit, Michigan, USA; 2 Department of Internal Medicine, Faculty of Medicine, American University of Beirut, Beirut, Lebanon

**Keywords:** pancreatitis, emergency medicine, disposition, therapy, Auxora, scoring system

## Abstract

Acute pancreatitis is a commonly encountered pathology in the emergency department. We presented a clinical review summarizing the contemporary emergency medicine approach to managing acute pancreatitis. Although the diagnostic criteria for acute pancreatitis are straightforward, it has many possible causes, several treatment options, and both short- and long-term sequelae. We discussed diagnostic, intervention, and disposition considerations relevant to emergency clinicians and considered risk assessment using available clinical decision tools. We also discussed changes to traditional treatments and ongoing investigational therapies, including steroids, monoclonal antibodies, and calcium release-activated calcium channel inhibitors.

## 1 Introduction

Acute pancreatitis (AP) is common in emergency medicine. It most commonly presents as interstitial pancreatitis (onset within a week, focal) and can progress to necrotizing pancreatitis (onset >7 days, with necrotic parenchyma and peripancreatic tissues). Short-term outcomes include pancreatic necrosis and respiratory failure, and long-term sequelae such as chronic pancreatitis (CP), diabetes mellitus, and pancreatic cancer.

Management strategies have changed over the past few decades, and new therapeutics are entering late-phase trials. In this state-of-the-art review, we defined the current epidemiology of AP, briefly discussed traditional considerations for diagnosing and treating it, and outlined advancements in diagnostic and management strategies.

## 2 Epidemiology

There has been an increase in the incidence of AP, with current data indicating a global incidence of 33 to 74 cases per 100,000 person-years.^1^, ^2^, ^3^ Males and females have similar rates of AP.^4^^,^^5^ The frequency of AP is markedly higher among Black patients compared with White, Hispanic, Asian, and Native American patients.^6^ The most common etiologies of AP include gallstones (40%), alcohol (30%),^7^ hypertriglyceridemia (5%), medications, trauma, postprocedural,^8^ and idiopathic pancreatitis.^9^

## 3 Emergency Department Evaluation

The classic presentation of AP includes acute, severe epigastric abdominal pain, often radiating to the back, with associated nausea and vomiting.^10^ In more severe cases, patients may have a fever, tachypnea, hypotension, diffuse, severe abdominal tenderness, and hypoactive bowel sounds.^11^^,^^12^

AP carries a broad differential, such as acute cholecystitis, bowel obstruction, peptic ulcer disease, or acute myocardial infarction.^13^ Immediate life-threatening conditions, such as myocardial infarction and perforated ulcers, should be ruled out as a priority.

**Figure 1:**
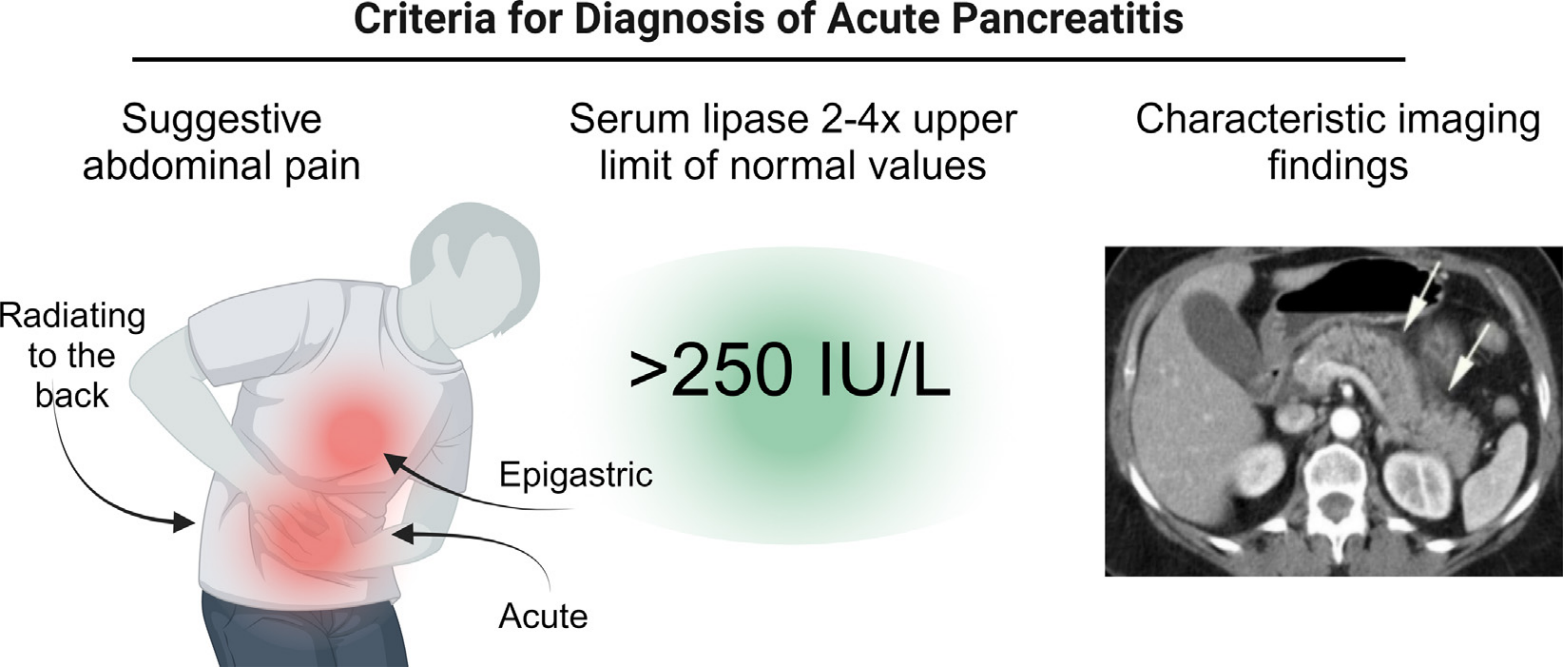
Criteria for acute pancreatitis diagnosis. (A) Acute, severe epigastric pain often radiates to the back. (B) Elevated lipase level of 2 to 4 times the upper limit of normal values. (C) Imaging consistently with interstitial pancreatitis. Created with BioRender.com.

The diagnosis of AP is made according to the revised Atlanta classification, which requires that at least 2 of 3 criteria be met (Fig 1).^14^ Suggestive symptomatology with diagnostic lipase levels in mild-to-moderate pancreatitis in stable patients is enough to nullify the need for imaging. Although diagnosing AP is often straightforward, investigating the etiology can be challenging but significantly aids early management decisions.

### 3.1 Laboratory Tests

Workup includes lipase and amylase levels.^15^ Disturbance in the secretion and continued production of pancreatic enzymes increases serum levels.^16^ Serum lipase level’s earlier increase, prolonged elevation, and better sensitivity make it the preferred test compared with serum amylase level.^15^^,^^17^ As such, amylase level testing is unnecessary in the emergency department (ED) evaluation of AP. Lipase level's wider diagnostic window makes it particularly helpful in patients presenting within the first 24 hours of symptoms or with delayed presentations.^17^ A lipase level 3 times or more than the upper limit of normal levels is suspicious for pancreatitis (normal lipase levels between 0 to 160 units/L or 0 to 2.67 microkat [μkat]/L).^18^^,^^19^

Additional testing considerations include a complete blood count and a complete metabolic panel (including alanine aminotransferase [ALT], lactate, C-reactive protein, and serum triglyceride levels).^10^ Evaluating triglyceride levels is particularly important in patients with diabetes or those without clear alcohol or gallstone AP. Evaluating the ALT level is useful in ruling out a biliary cause of AP. In a meta-analysis, ALT levels > 150 IU/L were associated with a 95% probability of gallstone pancreatitis.^20^

### 3.2 Imaging Studies

If a diagnosis of AP can be confirmed by characteristic abdominal pain and elevations in serum lipase level, abdominal imaging studies are not necessarily required for mild to moderate cases of AP, particularly when alcohol is a likely etiology of AP.^21^

In patients with noncharacteristic abdominal pain or serum pancreatic enzyme activities < 3 times the upper normal values, abdominal imaging with an intravenous contrast-enhanced computed tomography scan is required to establish the diagnosis.^22^^,^^23^ Imaging is also indicated for severe cases of AP (where systemic inflammatory response syndrome [SIRS] criteria are met), for suspected biliary pancreatitis, and when there is uncertainty regarding the etiology. Limited imaging is recommended when a strong diagnosis base exists to avoid reflex use of CT scans and unnecessary radiation exposure.

Transabdominal ultrasound is insensitive for diagnosing AP.^24^ Data show that contrast-enhanced abdominal ultrasound may have better test characteristics for diagnosing AP,^25^ but it requires additional expertise, and few centers use contrast for this indication.^26^ Despite these limitations, noncontrast transabdominal ultrasound can be a useful tool for assessing the gallbladder and biliary tree in managing suspected gallstone AP.^27^

In pregnant patients, ultrasound and magnetic resonance cholangiopancreatography (MRCP) are indicated for imaging diagnosis.^28^

### 3.3 Classification of Pancreatitis

Once a diagnosis of AP has been established, disease severity may be assessed using various classification strategies, most notably the revised Atlanta classification.^16^ This classification, revised in 2012, categorizes AP into 3 categories based on the presence of organ failure and local or systemic complications.^29^ In mild AP, there is no organ failure or local complications. In moderately severe AP, patients experience organ failure lasting <48 hours and may also experience local or systemic complications. In severe AP, patients experience single or multiple organ failure lasting >48 hours, with up to 20% mortality. Organ failure may be assessed using the modified Marshall scale (Table 1), which classifies failure as a score of ≥2 in the respiratory, cardiovascular, or renal systems.^22^

**Table 1:** Modified Marshall scale for assessing organ failure. The score incorporates the degree of lung, renal, and cardiovascular failure.

Parameter	Score				
	0	1	2	3	4
PaO_2_/FiO_2_ ratio	>400	301-400	201-300	101-200	≤101
Serum creatinine level (mg/dL)	<1.4	1.4-1.8	1.9-3.6	3.6-4.9	>4.9
Systolic blood pressure (mm Hg)†	>90	<90, fluid responsive	<90, not fluid responsive	<90, pH < 7.3	<90, pH < 7.2

A score of ≥2 in any system defines the presence of organ failure. The PaO_2_/FiO_2_ value can be estimated using SPO_2_ approximation for PaO_2_.

† Off ionotropic support.

### 3.4 Distinction from CP

CP is most commonly caused by alcohol use but may also be caused by smoking, genetic mutations, or recurrent episodes of AP.^30^ Clinical presentations of CP involve abdominal pain associated with anorexia, vomiting, weight loss, or steatorrhea.^31^ The similarities in the clinical presentations of AP and CP highlight the importance of thorough history-taking, with emphasis on any previous episodes of pancreatitis, gallbladder disease, alcohol abuse, or familial history of pancreatic disorders.

Diagnostic evaluation of CP relies more on history and imaging studies, as laboratory studies are nonspecific.^32^ Serum lipase levels may be only mildly elevated or normal.^32^ The preferred imaging modalities are CT or magnetic resonance imaging scans.^30^ In CP, images reveal pancreatic calcifications, atrophy, or ductal abnormalities.^33^^,^^34^ In patients with unclear etiologies of CP, referral for genetic mutations in the *PRSS1, SPINK1,* and *CTFR genes*, culprits of hereditary pancreatitis, can be considered.^35^

Ultimately, patients with CP can develop pancreatic fibrosis and subsequent loss of pancreatic endocrine and exocrine functions.^10^^,^^36^ CP is also associated with an increased risk of pancreatic cancer.^36^

## 4 Treatment

The ED management of patients with AP focuses on supportive care. Here we reviewed analgesic choices, parenteral or enteral fluids, and nutrition.

### 4.1 Analgesia

Data favoring a particular analgesic for AP-associated abdominal pain is lacking. Prior studies are limited and show no difference between diclofenac and tramadol^37^ or between paracetamol, dexketoprofen, and tramadol.^38^ Overall, studies have shown that non-narcotic pain control methods are non-inferior to opioids,^39^ suggesting that opioid-free analgesics should be used first. However, our experience suggests that parenteral opioids are often needed to manage pain associated with moderate to severe AP. A systematic review suggests that ketorolac could be a viable option for pain control and meeting management goals; however, further research should be performed to validate the conclusion.^40^

Opioid analgesia prescribing should be limited to as brief a treatment course as possible. Celiac block can be beneficial, albeit with low evidence for efficacy in AP.^41^, ^42^, ^43^ Ketorolac use is commonly effective, albeit with sparse clinical trial data.^40^

### 4.2 Parenteral Fluids

An early fluid replacement has been a cornerstone of managing AP, as the pathophysiology includes fluid third spacing in and around the pancreas.^18^ Crystalloid fluids are indicated in the ED, and medium-quality evidence favors Lactated Ringers (LR). Data suggested decreased systemic inflammation, intensive care unit (ICU) admission, and length of stay in patients administered LR compared to normal saline.^44^^,^^45^

Until recently, high-quality evidence was lacking to guide the volume of early resuscitation.^46^ Prior trials were underpowered but suggested potential harm with aggressive early fluid resuscitation.^47^ The recent WATERFALL trial provided high-quality evidence that early aggressive fluid resuscitation was inferior to a moderate approach. In this randomized controlled trial (RCT) of 249 patients with mild to moderate AP on enrollment, the authors compared aggressive (20 ml/kg bolus, then 3 mL/kg/h) to moderate fluid resuscitation (10 mL/kg bolus only in hypovolemic patients, then 1.5 mL/kg/h in all patients). The trial was stopped early due to a significantly higher incidence of fluid overload in those receiving aggressive therapy.^48^ It is notable, however, that the trial excluded patients who initially had signs of moderately severe or severe AP, such as shock, respiratory failure, or acute kidney injury.

Given the results of the WATERFALL trial, a strategy of moderate fluid resuscitation is appropriate for patients with mild-to-moderate AP. When patients in the ED have more severe disease from AP, it is unknown whether they may benefit from more aggressive fluid resuscitation. We suggest a guided approach to assessing clinical markers of adequate resuscitation, such as inferior vena cava ultrasonography or normalization of lactic acid levels. In addition to restoring perfusion to organs such as the kidneys, fluid resuscitation can potentially decrease AP necrosis by maintaining the microvasculature of the pancreas.^49^

### 4.3 Nil Per Os and Enteral Feeding

Although a widely accepted practice early in the treatment course for AP,^18^ NPO is contrary to current evidence and guidelines for the initial management of AP. Recent evidence demonstrates that enteral nutrition is associated with better outcomes within 48 hours of presentation.^50^ One RCT showed that a rapid, low-fat, solid diet, even in the ED, was not inferior to traditional, step-up nutritional advancements. Immediate initiation of a solid diet was associated with significant reductions in the length of hospital stays, ICU admissions, and complications.^51^ A trial comparing immediate to early oral feeding documented similar noninferiority and reduced the length of hospital stay.^52^ Another trial compared outcomes in children with AP receiving an experimental patient-directed diet to those of a retrospective group receiving standard care.^53^ The children in the group that received a patient-directed diet had a shorter length of hospital stay.^53^

A reasonable approach to enteral nutrition in the ED is to trial a solid diet when nausea and vomiting are controlled and patients are willing to attempt oral intake. In our experience, early oral tolerance in those with mild pancreatitis is often indicative of a safe discharge from the ED.

### 4.4 Emerging Therapies

**Figure 2:**
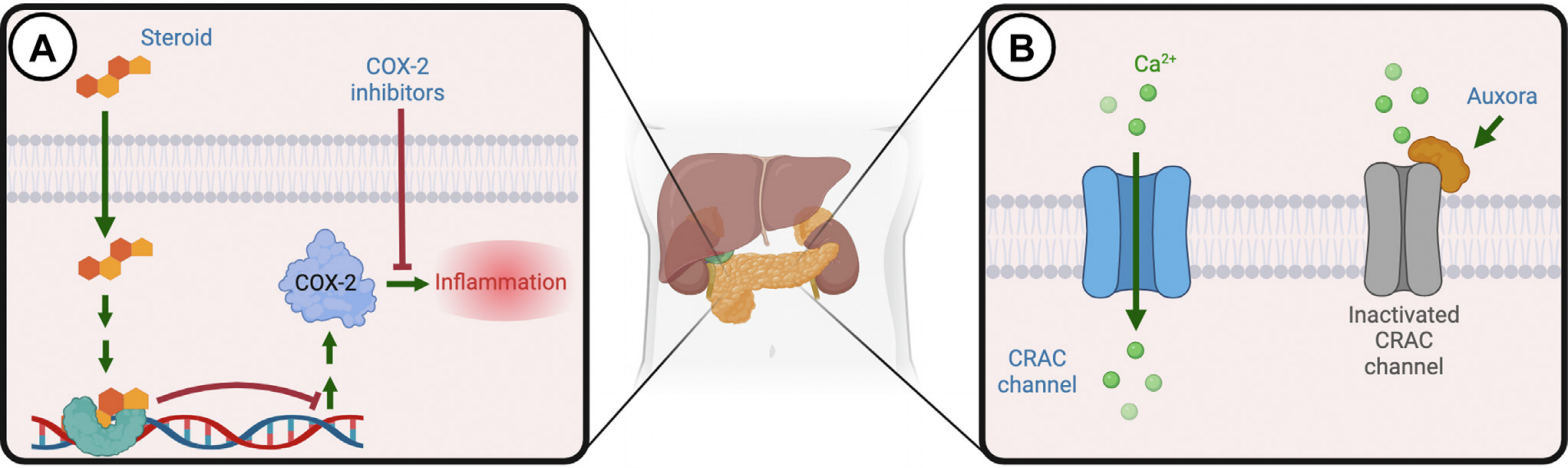
Mechanism of action of emerging therapies for acute pancreatitis. (A) It shows the classic intranuclear influence of steroids on reducing the transcription and expression of inflammatory markers. (B) It shows Auxora’s inhibition of CRAC channels, thereby blocking inflammatory cytokine release and reducing inflammation. CRAC, calcium release-activated calcium.

There are no US Food and Drug Administration (FDA)-approved medications for AP. We present potential emerging medications under active clinical investigation (Fig. 2). The first promising drug in late-phase development is Auxora, an injectable emulsion of CM4620. Auxora has a unique mechanism of inhibiting calcium release-activated calcium (CRAC) channels, which impacts inflammatory signaling and calcium regulation across cell membranes. In a phase 2, open-label trial, Auxora was associated with improved food tolerance and reduced hospitalization in patients with AP and severe inflammatory response syndrome.^54^ Auxora has demonstrated a favorable safety profile in AP and COVID-19 patients with severe illness.^55^^,^^56^ An ongoing clinical trial randomized 216 participants with AP to placebo or different doses of Auxora is ongoing.^57^

Corticosteroids and nonsteroidal anti-inflammatory drugs (NSAIDs) have been of interest in animal models for decades, given the associated inflammatory component to disease progression in AP.^58^^,^^59^ A study using selective cyclooxygenase 2 (COX-2) inhibitors (celecoxib and parecoxib) showed a lower incidence of severe AP in an animal model.^60^ Current guidelines recommend rectal administration of an NSAID shortly before the procedure to prevent postendoscopy retrograde cholangiopancreatography (ERCP) pancreatitis.^61^ Although clinical data on corticosteroid use in AP is scarce, an ongoing RCT is comparing corticosteroids (hydrocortisone) to placebo to reduce inflammation in severe AP cases.^62^

Lastly, 2 other immune-modulating medications are in human trials for AP. First, infliximab, a monoclonal antibody that binds tumor necrosis factor alpha (TNF-α), has shown preclinical evidence of reducing pancreatic necrosis. It is currently being tested in a phase 2 randomized controlled dose-finding trial.^63^ Second, pirfenidone, which is currently US FDA-approved for idiopathic pulmonary fibrosis, is an anti-inflammatory drug that decreases fibroblast proliferation. It, too, has shown promise in preclinical models.^64^ A clinical trial is underway in the US.^65^

## 5 Prognostic Tools in AP

The prognostication of AP has been extensively studied, and many tools are available.^49^ Nevertheless, most tools are intended for the ICU and inpatient setting and have not been tested for use in the ED.^66^ Widely used instruments have included Ranson’s criteria (specific to AP)^67^ and the broader acute physiology and chronic health evaluation II (APACHE-II) score, originally designed for the general risk stratification of critically ill patients. Likewise, the APACHE-II score is predictive of poor outcomes,^68^ but numerous elements are usually unavailable in the ED, limiting its overall utility.^69^

In contrast with the complexity of the APACHE-II, the bedside index of severity in acute pancreatitis (BISAP) score has comparable predictive accuracy for severe AP using a simple set of 5 criteria (blood urea nitrogen levels > 25 mg/dL, altered mental status, SIRS criteria > 2, age >60 years, presence of a pleural effusion).^70^ A score of ≥2 on the BISAP score has similar predictive power to the APACHE-II score in indicating a patient who may need critical care.^71^ Although useful for prognostication, the BISAP score instrument incorporates data from the first 24 hours of patient evaluation and is not explicitly validated for ED use. A more straightforward ED SPO_2_ (peripheral capillary oxygen saturation), age, and SIRS (ED-SAS) score incorporating SIRS, pulse oximetry, and age has been studied and validated in the ED and proven to be a prognostic of mortality.^54^ Data on the ED-SAS instrument showed that mortality rose significantly if patients had one or more criteria: age >60 years, pulse oximetry < 96%, or presence of ≥ 2 SIRS criteria. These scores share a common thread: age, pulmonary involvement, signs of systemic inflammatory response, and organ injury all predict significantly worse outcomes in AP.

## 6 Emergency Department Disposition and Consultation

**Figure 3:**
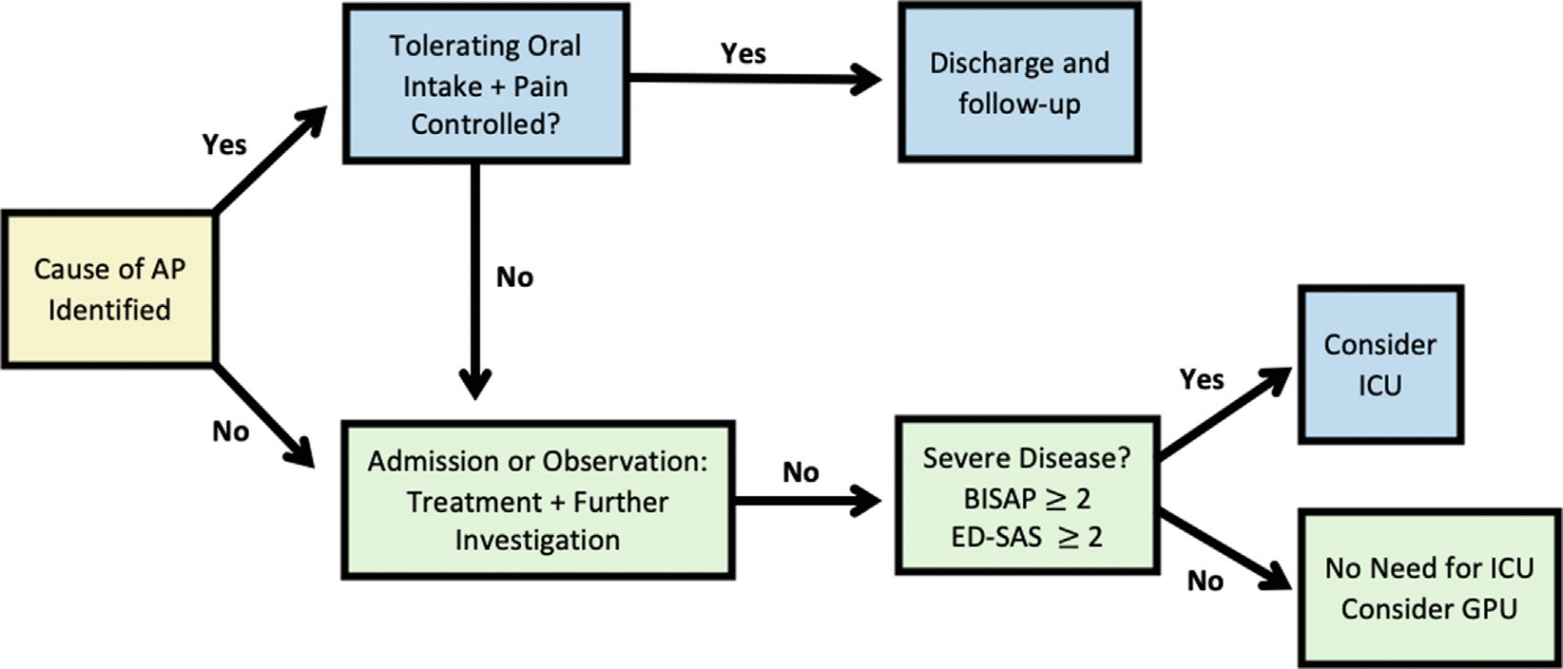
Considerations for disposition from the emergency department. AP, acute pancreatitis; BISAP, bedside index of severity in acute pancreatitis; ED-SAS, emergency department SPO_2_, Age, and systemic inflammatory response syndrome score; GPU, gastroenterology procedure unit; ICU, Intensive care unit; SPO_2,_ oxygen saturation.

For patients with mild AP, the decision regarding hospitalization or outpatient management is mainly guided by oral tolerance, severity of abdominal pain, and the suspected etiology (Figure 3). Patients who can tolerate oral intake, have manageable pain, and have a suspected etiology that does not require immediate intervention may be considered for ED discharge. Conditions such as pancreatic duct calculus or severe hypertriglyceridemia may require hospitalization regardless of AP severity. Mild cases due to alcohol, medications, or post-ERCP may often be appropriate for ED discharge or a period of observation placement. Patients with AP due to suspected alcohol abuse could benefit from a screening, brief intervention, referral, and treatment approach to improve alcohol cessation.

### 6.1 Consultation and Transfer

Surgical or gastroenterology consultation is frequently needed in managing patients with AP, particularly when history and imaging suggest gallstone pancreatitis. Several studies have shown that early cholecystectomy is better than delayed or referred cholecystectomy for patients with biliary causes of AP, as long as the patient is stable and the surgery can be done.^72^, ^73^, ^74^, ^75^

Further diagnostic testing with ERCP or MRCP is rarely necessary for the early evaluation of AP. When there is a suspicion of concomitant cholangitis, an urgent ERCP is necessary to confirm the diagnosis and provide biliary drainage.^76^ In these cases, transfer to sites with interventional gastroenterology expertise may be required.

Local AP complications encountered in the ED will occasionally require early specialty consultation and transfer when appropriate expertise is unavailable. Acute peripancreatic fluid collections are common and usually resolve without drainage. The absence of a well-defined wall characterizes these fluid collections, which do not require surgical consultation. Pancreatic pseudocysts, on the other hand, have well-defined walls. Pseudocysts are usually a late complication of AP and are less commonly present on ED imaging. When asymptomatic, it is reasonable to observe pancreatic pseudocysts. When symptoms are present, such as pain, vomiting, jaundice, or fever, management requires a multidisciplinary approach, including expertise from gastroenterology, interventional radiology, and surgery consultants.

Additional considerations for surgical consultation include the presence of perforating pancreatitis (such as a perforating duodenal ulcer), traumatic pancreatitis, and rapidly deteriorating cases of AP.

Table 2 summarizes these considerations and other take-home points in the ED management of AP.

**Table 2:** Take-home points for emergency medicine management of AP.

Diagnosis	Symptoms and lipase level elevation define the diagnosis. If there is an unclear diagnosis or severe pancreatitis, imaging is recommended.	
Severity	Physiologic derangements and organ injury indicate severity. Consider the use of BISAP or ED-SAS scores in the ED.	
Therapy	Pain control	Opiate sparing strategy for mild AP. Judicious opiate as needed for moderate to severe AP.
	Fluid Resuscitation	Moderate (10 mL/kg bolus only in hypovolemic patients, then 1.5 mL/kg/h) was associated with better outcomes than aggressive fluid resuscitation.
	Feeding	Early initiation (even in the ED) as early as tolerated may be associated with better outcomes.
	Investigational therapies	Auxora, infliximab, pirfenidone, and others in development.
Consultation	Driven by etiology and associated complications of AP. Cholecystectomy is indicated during hospitalization for biliary etiology of AP.	

AP, acute pancreatitis; BISAP, bedside index of severity in acute pancreatitis; ED, emergency department; ED-SAS, emergency department SPO_2_, Age, and systemic inflammatory response syndrome score.

### 6.2 Follow-up

Clinicians should refer patients with AP to primary care or gastroenterology for ongoing care after their discharge from the ED or observation unit. Besides symptom control, follow-up care may include additional testing for rare causes of AP when the etiology is unclear in the ED and monitoring for long-term complications such as pancreatic insufficiency, postpancreatic diabetes, and chronic pancreatitis.^77^ Approximately a third of patients with recurrent AP will develop chronic pancreatitis.^78^ For patients with alcohol-induced AP, alcohol counseling and referral to mutual help groups or behavioral therapy are recommended to prevent further episodes of the condition.

Other aspects of follow-up may depend on the unique etiology of AP. Patients with biliary etiologies require surgical follow-up to consider cholecystectomy when it was not performed during their hospitalization.^73^^,^^74^ Patients with hypertriglyceridemia-induced pancreatitis require continuous endocrinological monitoring and dyslipidemia treatment.

## 7 Conclusion

In the ED, prompt diagnosis, risk assessment, management, consultation, and disposition of patients with AP are paramount to reducing short- and long-term complications. Research within AP management, such as AP-specific analgesic options and novel immune-modulating therapies, may offer advances in AP management and improve patient comfort and outcomes.

## Author Contributions

JM conceived the study. KH, MS, TH, SC, and CC designed and drafted the manuscript. JM provided clinical insights and supervised manuscript writing. All authors contributed to critical review and revision of the paper.

## Funding and Support

By *JACEP Open* policy, all authors are required to disclose any and all commercial, financial, and other relationships in any way related to the subject of this article as per ICMJE conflict of interest guidelines (see www.icmje.org). The authors have stated that no such relationships exist.

## Conflict of Interest

All authors have affirmed they have no conflicts of interest to declare.
